# Identification of MRP2 as a targetable factor limiting oxaliplatin accumulation and response in gastrointestinal cancer

**DOI:** 10.1038/s41598-019-38667-8

**Published:** 2019-02-19

**Authors:** Khine Myint, Riya Biswas, Yan Li, Nancy Jong, Stephen Jamieson, Johnson Liu, Catherine Han, Christopher Squire, Fabrice Merien, Jun Lu, Takeo Nakanishi, Ikumi Tamai, Mark McKeage

**Affiliations:** 1 Department of Pharmacology and Clinical Pharmacology, University of Auckland, Auckland, New Zealand; 2 AUT-Roche Diagnostics Laboratory, School of Science, Auckland University of Technology, Auckland, New Zealand; 3 School of Interprofessional Health Studies, Auckland University of Technology, Auckland, New Zealand; 4 Auckland Cancer Society Research Centre, University of Auckland, Auckland, New Zealand; 5 Department of Pharmacology, School of Medical Sciences, University of New South Wales, Sydney, NSW 2052 Australia; 6 School of Biological Sciences, University of Auckland, Auckland, New Zealand; 7 Department of Membrane Transport and Biopharmaceutics, Faculty of Pharmaceutical Sciences, Institute of Medical, Pharmaceutical and Health Sciences, Kanazawa University, Kakuma-machi, Kanazawa, 920-1192 Japan

## Abstract

Oxaliplatin is important for the clinical treatment of colorectal cancer and other gastrointestinal malignancies, but tumour resistance is limiting. Several oxaliplatin transporters were previously identified but their relative contributions to determining oxaliplatin tumour responses and gastrointestinal tumour cell sensitivity to oxaliplatin remains unclear. We studied clinical associations between tumour expression of oxaliplatin transporter candidate genes and patient response to oxaliplatin, then experimentally verified associations found with MRP2 in models of human gastrointestinal cancer. Among 18 oxaliplatin transporter candidate genes, MRP2 was the only one to be differentially expressed in the tumours of colorectal cancer patients who did or did not respond to FOLFOX chemotherapy. Over-expression of MRP2 (endogenously in HepG2 and PANC-1 cells, or induced by stable transfection of HEK293 cells) decreased oxaliplatin accumulation and cytotoxicity but those deficits were reversed by inhibition of MRP2 with myricetin or siRNA knockdown. Mice bearing subcutaneous HepG2 tumour xenografts were sensitised to oxaliplatin antitumour activity by concurrent myricetin treatment with little or no increase in toxicity. In conclusion, MRP2 limits oxaliplatin accumulation and response in human gastrointestinal cancer. Screening tumour MRP2 expression levels, to select patients for treatment with oxaliplatin-based chemotherapy alone or in combination with a MRP2 inhibitor, could improve treatment outcomes.

## Introduction

Chemotherapy with the platinum-based drug oxaliplatin is of major importance for the clinical treatment of colorectal cancer and other gastrointestinal malignancies. Colorectal cancer and the other gastrointestinal malignancies treatable by oxaliplatin-based chemotherapy are among the most common cancer types and causes of cancer death in the world today^1^. Robust clinical evidence of the efficacy of oxaliplatin-based chemotherapy from well-designed randomised controlled trials have shown improved patient outcomes in colorectal cancer, both in the adjuvant^2^ and metastatic settings^3,4^, and in pancreatic^5,6^, oesophagogastric^7,8^ and hepatocellular^9^ cancer. Although oxaliplatin-based chemotherapy has been widely adopted as the standard and preferred chemotherapy regimen for treating many types of gastrointestinal cancer^10,11^, its toxicity and resistance are major clinical limitations.

Oxaliplatin must cross cell membranes before causing cytotoxicity in tumour cells by reacting with DNA and forming DNA–platinum adducts that induce cell cycle arrest and cell death^12^. Oxaliplatin’s inherent capacity for crossing cell membranes by passive diffusion may be limited by its hydrophilicity^13,14^ and chemical transformation into charged intermediates in biological fluids^15^. Over the last decade, evidence has accumulated for membrane transporter proteins controlling the movement of oxaliplatin into and out of cells^16^. Several membrane transporter proteins from the ATP binding cassette (*ABC*), solute carrier (*SLC*) and ATPase membrane protein superfamilies have been reported to transport oxaliplatin *in vitro*, in tumour lines selected for oxaliplatin resistance or in cells genetically modified to alter expression of individual membrane transporter genes (Table 1). However, the relative contributions of specific membrane transporters to determining gastrointestinal tumour clinical responses to oxaliplatin-based chemotherapy remains unclear.

**Table 1:** Oxaliplatin transporter candidate genes. Adapted and updated from^16^. References are the original reports of oxaliplatin transport by each transporter.

Super family	Gene	Reference
*ABC*	*ABCC1*/MRP1	^30^
	*ABCC2*/MRP2	^24– 26, 36^
	*ABCC4*/MRP4	^30^
*SLC*	*SLC21A6*/OATP1B3	^49^
	*SLC22A1*/OCT1	^50– 52^
	*SLC22A2*/OCT2	^51– 55^
	*SLC22A3*/OCT3	^54– 56^
	*SLC22A4*/OCTN1	^57^
	*SLC22A5*/OCTN2	^57^
	*SLC22A6*/OCT6	^58^
	*SLC31A1*/CTR1	^59– 62^
	*SLC47A*/MATE1/2-K	^54, 55^
ATPases	*ATP7A*	^63– 66^
	*ATP7B*	^63– 65^

Multidrug resistance-associated protein 2 (MRP2) is a 190 kDa glycoprotein encoded by the *ABCC2* gene, which functions to transport a range of substrates across cell membranes using energy derived from ATP hydrolysis^17^. MRP2 is highly expressed in the normal gastrointestinal system, for example, on the apical membranes of colonic enterocytes and biliary canalicular membranes of hepatocytes, where it functions in the excretion of substances into the gut lumen and bile^17^. Some tumour cells also express MRP2, including colorectal, hepatocellular and other gastrointestinal cancer cells, in which MRP2 can confer multidrug resistance by virtue of its function as a poly-specific drug efflux pump^17^. Earlier work established MRP2 as an efflux transporter of cisplatin and mediator of cisplatin resistance^18–22^. However, there have been few studies of the influence of MRP2 in oxaliplatin therapy of gastrointestinal cancer^23–26^ despite its major therapeutic role in this clinical setting.

With this background, we carried out the study described here with the aim of identifying membrane transporter proteins that determine clinical sensitivity of human gastrointestinal cancer to oxaliplatin. First, we examined clinical associations between the tumour expression of oxaliplatin transporter candidate genes and patient response to oxaliplatin-based chemotherapy. Then, we experimentally verified the major clinical association found with MRP2 in models of human gastrointestinal cancer. In these *in vitro* and *in vivo* experimental systems, the expression and activity of MRP2 was manipulated by siRNA gene knockdown and pharmacological inhibition with a model compound (myricetin)^27,28^ that had low potential for reaction with platinum compounds.

## Results

### Clinical association

**Figure 1:**
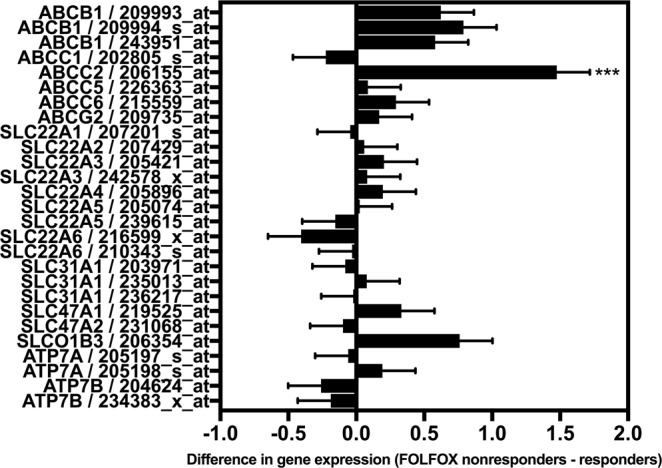
Differential expression of oxaliplatin transporter candidate genes in colorectal tumours of patients who responded or did not respond to FOLFOX chemotherapy. Data from the Tsuji Colorectal dataset^29^ from Oncomine included 83 colorectal cancer patients who had tumour microarray gene expression analysis prior to FOLFOX chemotherapy then stratification by tumour response. Bars and error bars are means and standard errors of differences in the expression of individual oxaliplatin transporter candidate genes between responding and non-responding patients. Positive differences indicate higher expression in non-responders whereas negative differences indicate higher expression in responders. The asterisk is a *P* value (***P < 0.0001) from a Bonferroni post-test that followed a Two-way ANOVA (Two-way ANOVA Factors: Tumour response, *P* = 0.0025; Gene, *P* < 0.0001; Interaction, *P* = 0014). *ABCC2*/MRP2 was over-expressed in colorectal tumours from patients who did not respond to FOLFOX chemotherapy compared to responders. Other oxaliplatin transporter candidate genes were expressed similarly in both responding and non-responding patients.

MRP2 was significantly overexpressed in the colorectal tumours of patients who did not respond to oxaliplatin chemotherapy. We searched the Oncomine transcriptome database for datasets of patients treated with oxaliplatin, who had tumour microarray gene expression profiling undertaken before treatment and annotation of their subsequent tumour response. Only one dataset was found, the Tsuji Colorectal dataset^29^ (GDS4393 and GDS4396) comprising of 83 patients with metastatic colorectal cancer who had tumour microarray gene expression profiling before treatment with FOLFOX. Patients were stratified into FOLFOX responders (n = 42) or non-responders (n = 41). Differences between the two groups in the expression of reporters of each oxaliplatin transporter candidate gene (Table 1) were calculated. Only one of 18 oxaliplatin transporter candidate genes showed significantly different expression. MRP2 (*ABCC2*) was overexpressed (1.5-fold) in the tumours of patients who did not respond (*P* < 0.0001 Bonferroni post-test following Two-Way ANOVA; Two-way ANOVA Factors: Tumour response, *P* = 0.0025; Gene, *P* < 0.0001; Interaction, *P* = 0014) (Fig. 1). The tumour expression of the other 17 oxaliplatin transporter candidate genes showed no significant difference (<−0.4- to 0.8-fold) between responding and non-responding patients.

### *In vitro* studies

**Figure 2:**
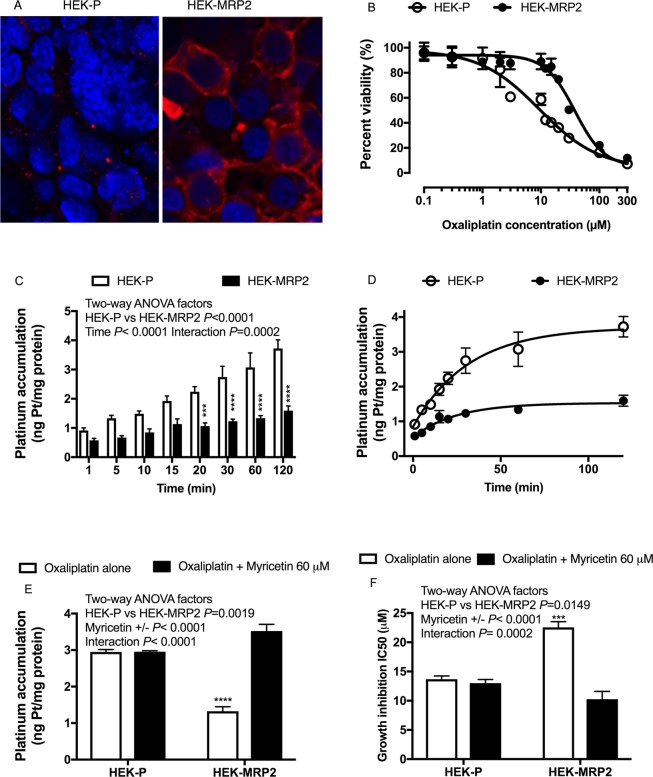
Oxaliplatin accumulation and cytotoxicity in HEK293 cells stably transfected to overexpress MRP2 (HEK-MRP2 cells) compared to control cells (HEK-P cells). (**A**) MRP2 was localised to the plasma membranes of HEK-MRP2 cells as demonstrated by MRP2-immunofluorescence confocal microscopy. Red staining, MRP2; Blue staining, DAPI. (**B**) Representative inhibition of growth of HEK-MRP2 and HEK-P cells after 2-hour exposure to oxaliplatin. Symbols are means and standard errors of the mean [n = 4]. Solid lines are non-linear regression fits (Y = Bottom + (Top − Bottom)/(1 + 10^((LogIC50 − X)*HillSlope))^) to the data (r^2^ > 0.97). (**C**) Time-course of platinum accumulation during exposure to oxaliplatin for up to two hours showing lower accumulation of platinum in HEK-MRP2 cells. Asterisks are *P* values (***P < 0.001; ****P < 0.0001) for differences at each time point from Bonferroni post-tests that followed a Two-way ANOVA [n = 6]. (**D**) Kinetics of platinum accumulation during exposure to oxaliplatin for up to two hours showing a plateau in levels after one-hour exposure. Solid lines represent non-linear regression fits (y = ymax(1− e^−kx^) to the data (r^2^ > 0.92). (**E,F**) Effect of myricetin (60 μM) on oxaliplatin accumulation [n = 3] (E) and cytotoxicity [n = 3–4] (F). Asterisks are *P* values (***P < 0.001; ****P < 0.0001) for differences with each of the other three groups from Bonferroni post-tests that followed a Two-way ANOVA. Bars represent means and standard errors of the mean. HEK-MRP2 cells accumulated less platinum and were less sensitive to oxaliplatin-induced growth inhibition than control HEK-P cells. Myricetin increased the accumulation and cytotoxicity of oxaliplatin in HEK-MRP2 cells but not in HEK-P cells.

In an isogenic pair of HEK293 cell lines, stable overexpression of MRP2 (HEK-MRP2 cells) decreased oxaliplatin accumulation and cytotoxicity but those deficits were reversed by inhibition of MRP2 with myricetin. Immunofluorescence confocal microscopy detected MRP2 protein localised to the plasma membranes of HEK-MRP2 cells but negligible immunoreactivity in parental HEK cells (HEK-P cells) (Fig. 2A). Compared to HEK-P cells, HEK-MRP2 cells accumulated up to 3-fold less platinum during exposure to oxaliplatin for up to 2 hours (Fig. 2C,D). Concurrent exposure to oxaliplatin with myricetin increased the accumulation of platinum in HEK-MRP2 cells by about 3-fold (*P* < 0.001) but did not change the accumulation of platinum in HEK-P cells (Fig. 2E). IC_50_ values for oxaliplatin-induced growth inhibition were increased in HEK-MRP2 cells by about 2-fold compared to HEK-P cells (Fig. 2B,F). Concurrent exposure to oxaliplatin with myricetin increased the sensitivity of HEK-MRP2 cells to oxaliplatin-induced growth inhibition but had no effect on HEK-P cells (Fig. 2F).

**Figure 3:**
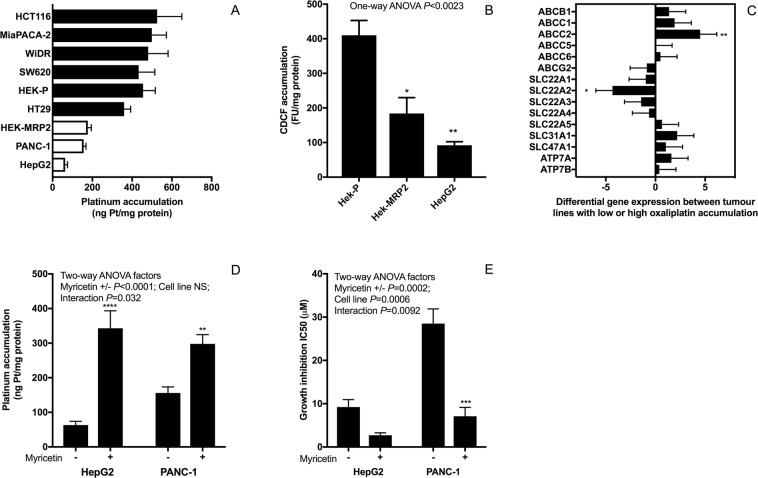
Identification of human gastrointestinal tumour lines with MRP2-mediated deficits in oxaliplatin accumulation. (**A**) Platinum accumulation after exposure to oxaliplatin (50 μM) for 2 h showing lower platinum accumulation in HepG2 and PANC-1 cells (open bars) compared to other tumour lines (closed bars) [n = 4]. (**B**) CDCF accumulation in HEK-P, HEK-MRP2 and HepG2 cells. Asterisks are *P* values (*P < 0.05; **P < 0.01) from Bonferroni post-tests that followed One-way ANOVA for comparisons to HEK-P cells [n = 3]; (**C**) Differential expression of oxaliplatin transporter candidate genes between tumour lines with high (6 cell lines) versus low (3 cell lines) oxaliplatin accumulation (closed versus open bars from panel (**A**)) determined by transporter real-time PCR array analysis. Positive differences indicate higher expression in lines with lower oxaliplatin accumulation whereas negative differences indicate higher expression in lines with higher oxaliplatin accumulation. Asterisks are *P* values (*P < 0.05; **P < 0.01) from Bonferroni post-tests that followed Two-way ANOVA (Two-way ANOVA factors: Gene *P* < 0.0001; Platinum accumulation low versus high, NS; Interaction, NS) [n = 3 replicates per cell line]. (**D**,**E**) Effect of myricetin (60 μM) on the accumulation (**D**) [n = 4–5] and cytotoxicity (E) [n = 3] of oxaliplatin in HepG2 and PANC1 cells. Asterisks are *P* values (**P < 0.01; ***P < 0.001; ****P < 0.0001) from Bonferroni post-tests that followed Two-way ANOVA. HepG2 and PANC-2 cells accumulated less oxaliplatin compared to other GI tumour lines. Inhibition of MRP2 by myricetin increased the accumulation and cytotoxicity of oxaliplatin in HepG2 and PANC-1 cells. Bars are the means and standard errors of the mean.

To identify clinically relevant experimental models, two human gastrointestinal tumour lines (HepG2 and PANC-1) were identified as having MRP2-mediated deficits in oxaliplatin accumulation reversed by inhibition of MRP2 with myricetin or siRNA *ABCC2* gene knockdown. Like HEK-MRP2 cells, HepG2 and PANC1 cells accumulated less platinum than HEK-P cells during *in vitro* exposure to oxaliplatin (50 μM) for two hours (Fig. 3A). In contrast, other human gastrointestinal cancer lines (HCT116, MiaPACA-2, WiDr, SW620, and HT29) accumulated similar levels of platinum as HEK-P cells but more than by HEK-MRP2, HepG2 and PANC-1 cells (Fig. 3A). HepG2 cells accumulated less model MRP2 substrate (5(6)-carboxy-2,′7′-dichlorofluorescein (CDCF)) than HEK-P cells, as did the HEK-MRP2 cells (Fig. 3B). Real-time PCR array analysis of transporter gene expression revealed higher expression of MRP2 and lower expression of OCT2 (*SLC22A2)* in tumour lines with reduced accumulation of oxaliplatin (HepG2, PANC-1, HEK-MRP2) (Fig. 3C). Concurrent exposure to oxaliplatin with myricetin increased the accumulation and cytotoxicity of oxaliplatin, by 1.9 to 5.4-fold and 3.4 to 4.0-fold, respectively, in both HepG2 and PANC-1 cells (Fig. 3D,E).

**Figure 4:**
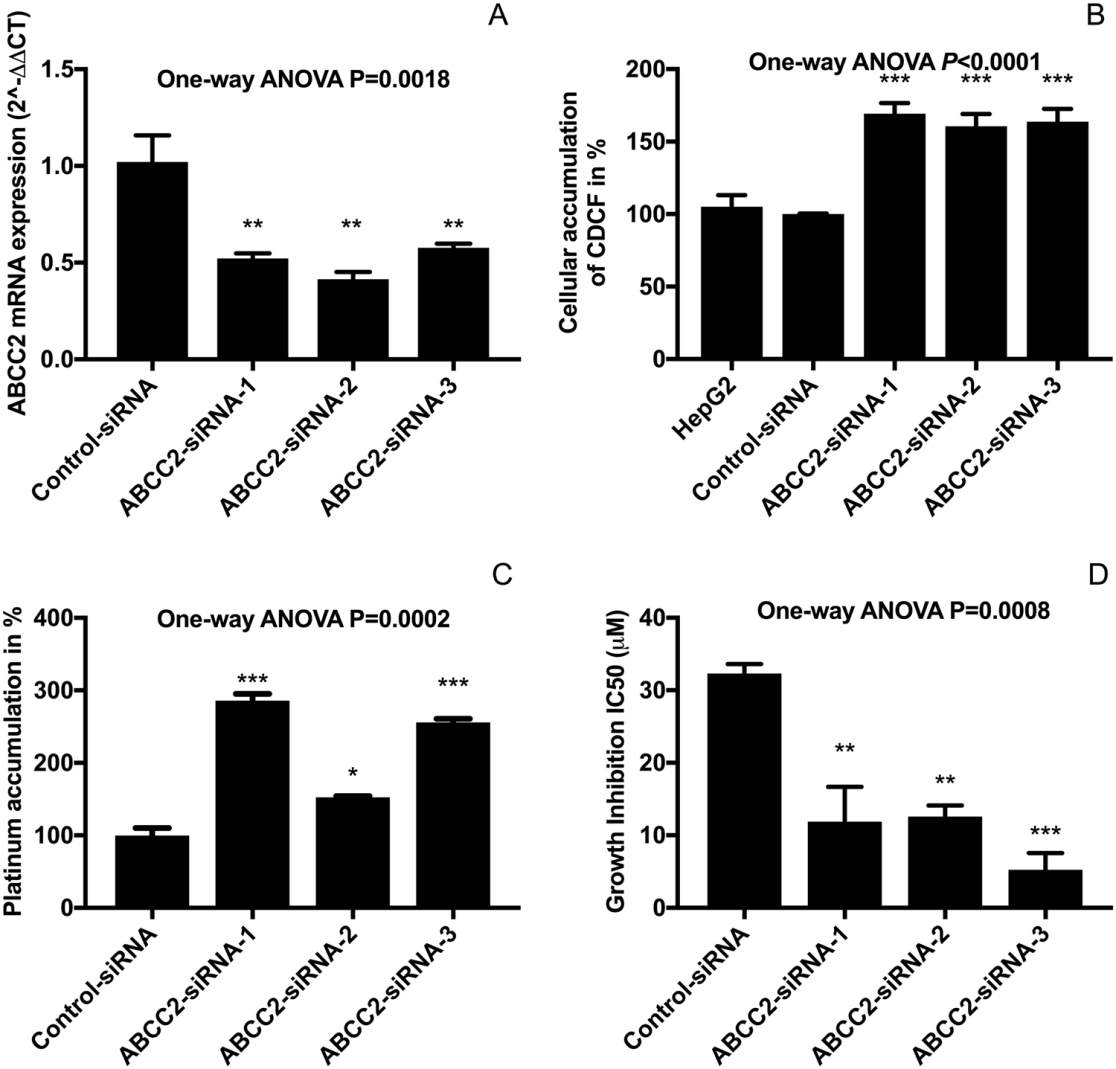
Effect of *ABCC2* siRNA gene knockdown on MRP2 mRNA expression (**A**), CDCF accumulation (**B**), oxaliplatin accumulation (**C**) and oxaliplatin-induced growth inhibition (**D**) in HepG2 cells. Asterisks are *P* values (*P < 0.05; **P < 0.01; **P < 0.001) from Bonferroni post-tests that followed one-way ANOVA. Inhibition of MRP2 by siRNA gene knockdown increased the accumulation and cytotoxicity of oxaliplatin in HepG2 cells. Bars are the means and standard errors of the mean (n = 3).

MRP2 gene knockdown significantly increased the cellular accumulation of platinum and enhanced the sensitivity to oxaliplatin in HepG2 cells. HepG2 cells were transfected with three independent MRP2-siRNAs and negative control siRNA using Lipofectamine RNAiMAX. The expression of MRP2 mRNA was significantly decreased in MRP2-siRNA transfected HepG2 cells compared with controls (Fig. 4A). Silencing MRP2 led to increase of cellular accumulation of an MRP2 substrate, CDCF by 160% to 170% (Fig. 4B). The cellular platinum accumulation after 2 h exposure to 25 µM oxaliplatin in control HepG2 cells was 40.9 ± 5.9 pmol per mg protein. Compared with the controls, platinum accumulation in siRNA treated HepG2 cells was significantly higher (117 ± 5.3 (*P* < 0.001), 62.4 ± 1.0 (*P* < 0.05), 104.7 ± 2.9 (*P* < 0.001) pmol pt/mg protein in siRNA-1, siRNA-2 and siRNA-3; respectively) (Fig. 4C). IC_50_ values against oxaliplatin were 2- to 5-fold more potent in HepG2 cells transfected with control siRNA compared with those transfected with MRP2-siRNAs (Fig. 4D).

### *In vivo* study

**Figure 5:**
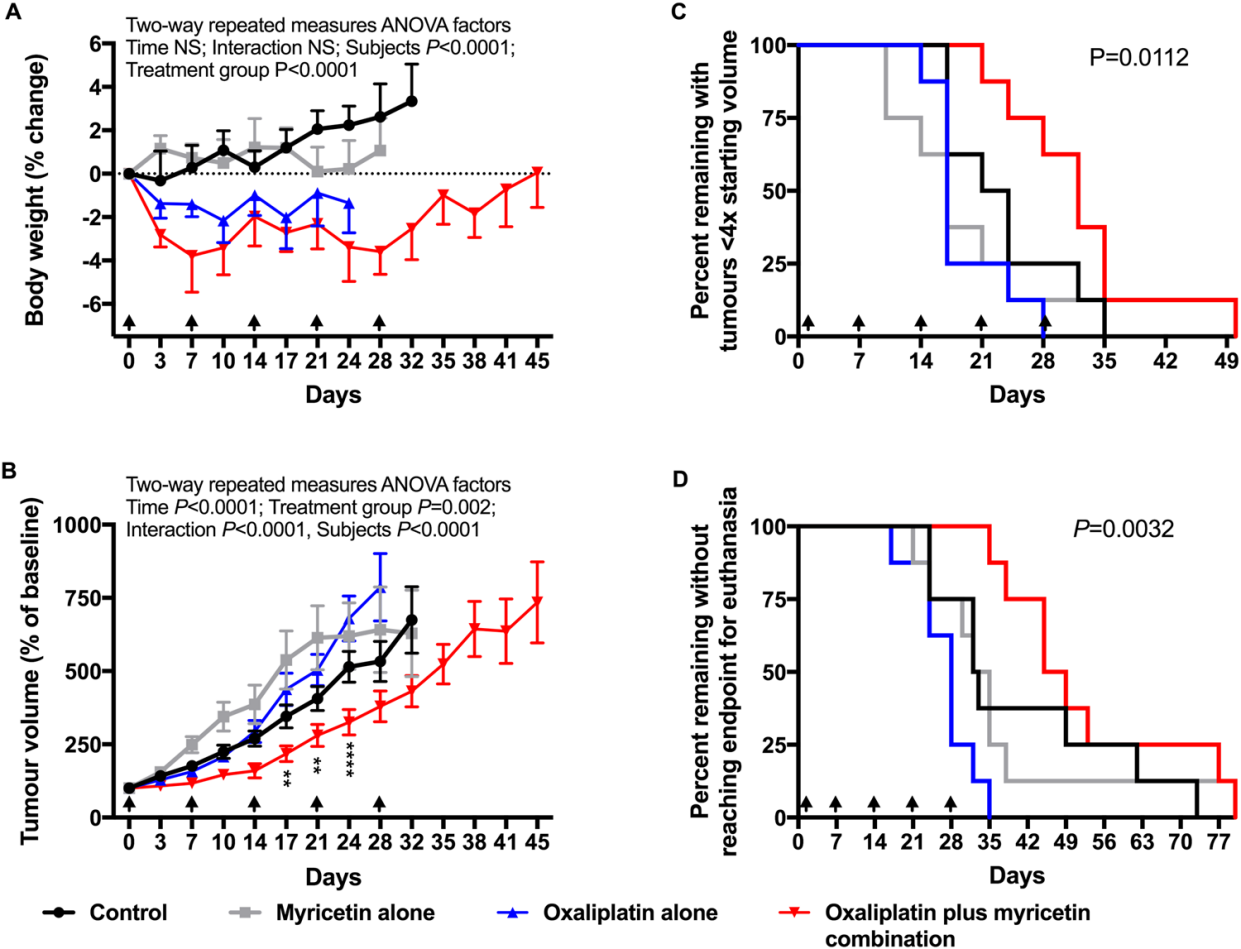
*In vivo* sensitisation to oxaliplatin antitumour activity by inhibition of MRP2 with myricetin. Nude mice (NIH-III) were implanted subcutaneously with HepG2 cells, which showed MRP2-mediated deficits in oxaliplatin accumulation *in vitro*. Tumour bearing mice (8 per group) were then treated with oxaliplatin (3 mg/kg ip), myricetin (25 mg/kg iv) or matching drug vehicle, alone or in combination once weekly for five weeks starting when tumours measured approximately 200 mm^3^. Arrowheads are the time of treatment. Experimental endpoints measured during and after treatment included body weight (**A**), tumour volume (**B**), time for tumours to quadruple in volume from baseline (**C**), and time to euthanasia from the start of treatment (**D**). Asterisks shown in panel B are *P* values (***P < 0.002; ****P < 0.0001) for differences between the oxaliplatin alone and oxaliplatin plus myricetin treatment groups at each time point from Bonferroni post-tests that followed Two-way Repeated Measures ANOVA. *P* values shown in panels C and D are for comparisons of all treatment groups. Oxaliplatin reduced body weight but had no antitumour activity when given alone. When combined with myricetin, oxaliplatin slowed tumour growth and extended the survival of animals with little or no increase in toxicity.

Mice bearing subcutaneous HepG2 tumour xenografts were sensitised to the *in vivo* antitumour activity of oxaliplatin by concurrent treatment with myricetin with little or no increase in toxicity. Nude mice (NIH-III) were implanted subcutaneously with HepG2 cells, which were demonstrated to have MRP2-mediated deficits in oxaliplatin accumulation *in vitro*. Tumour bearing mice were treated with oxaliplatin (3 mg/kg ip), myricetin (25 mg/kg iv) or their respective vehicles alone or in combination (n = 8/group), once weekly for five weeks, starting from when tumours measured about 200 mm^3^ (range, 161 to 237 mm^3^). The body weight of mice was reduced by oxaliplatin treatment when given alone or in combination with myricetin, compared to drug vehicle or myricetin alone (Repeated Measures Two-Way ANOVA Factors: Time, NS; Treatment group, *P* < 0.0001; Interaction, NS; Subjects, *P* < 0.0001) (Fig. 5A and Supplementary Table 1). Growth of HepG2 tumour xenografts was slowed by treatment with oxaliplatin combined with myricetin compared to treatment with drug vehicle alone, oxaliplatin alone or myricetin alone (Repeated Measures Two-Way ANOVA Factors: Time, *P* < 0.0001; Treatment group, *P* = 0.002; Interaction, *P* < 0.0001; Subjects, *P* < 0.0001)(Fig. 5B and Supplementary Table 2). The time taken for HepG2 tumours to quadruple in size was prolonged by treatment with oxaliplatin combined with myricetin (median, 32 days) compared to drug vehicle alone (median, 22.5 days; *P* = 0.0454), oxaliplatin alone (median, 17 days; *P* = 0.0016) or myricetin alone (median, 17 days; *P* = 0.015)(*P* value for comparison of all groups = 0.0112) (Fig. 5C and Supplementary Table 3). The time taken to reach the endpoint of euthanasia was prolonged by treatment with oxaliplatin combined with myricetin (median, 47 days) compared to drug vehicle alone (median, 33.5 days; *P* = 0.0968), oxaliplatin alone (median, 28 days; *P* = 0.0003) or myricetin alone (median, 17 days; *P* = 0.0181)(*P* value for comparison of all groups = 0.0032) (Fig. 5D and Supplementary Table 3).

## Discussion

Oxaliplatin and its combination regimens are of major importance in the clinical treatment of colorectal cancer and other gastrointestinal malignancies, but some patients fail to respond to treatment. National and international clinical practice guidelines^10,11^ now recommend oxaliplatin-based chemotherapy as a preferred regimen for the treatment of colon, gastric, oesophageal, pancreatic, rectal and other types of gastrointestinal cancer based on robust evidence from multiple randomised controlled trials^2–9^. However, in some patients the best tumour response is progressive disease and in others tumour responses are short-lived. Currently it is not possible to prospectively identify individual patients who are destined to fail oxaliplatin therapy prior to their exposure to treatment and observation of their unsatisfactory clinical outcomes. Moreover, there is an urgent need to identify alternative treatments for patients who will have such poor outcomes from standard oxaliplatin-based chemotherapy.

In the current study, we demonstrated a significant clinical association between tumour expression of the oxaliplatin transporter MRP2 and lack of responsiveness of colorectal cancer patients to standard oxaliplatin-based chemotherapy (FOLFOX). In contrast, tumour expression of 17 other oxaliplatin transporter candidate genes showed no significant association with clinical response to oxaliplatin in this dataset. Unfortunately, only one dataset was available in Oncomine of gastrointestinal cancer patients with tumour gene expression profiling undertaken before treatment with oxaliplatin together with annotation of their subsequent tumour response. Moreover, in this dataset, only steady state MRP2 mRNA levels were measured, not MRP2 protein level or function, which may be altered by posttranslational modification, such as by glycosylation^30,31^. However, these data we now report add to an increasing number of independent studies reporting clinical associations between gastrointestinal tumour expression of MRP2 and non-responsiveness to chemotherapy with oxaliplatin or cisplatin^23,32–35^. Taken together, these findings suggest that MRP2 may cause clinical resistance to oxaliplatin in gastrointestinal cancer patients, but ultimate proof of a causal association will require generation and interrogation of other tumour gene expression clinical datasets along with prospective clinical trials evaluating the inhibition of MRP2 in combination with oxaliplatin in patients whose tumours overexpress MRP2.

Our *in vitro* experimental studies reported here provided insights into mechanisms underlying poor responses to oxaliplatin in gastrointestinal cancer. The mechanism identified here involves MRP2 actively transporting oxaliplatin and/or other cytotoxic platinum species derived from oxaliplatin, resulting in their extrusion from tumour cells, net reduction in cellular platinum accumulation and decreased inhibitory effects of oxaliplatin on cell growth. Previously, we demonstrated that MRP2 actively transports oxaliplatin-derived platinum in MRP2-expressing membrane vesicles^36^. Intact oxaliplatin and/or its anionic monochloro oxalate ring-opened early degradation product were identified in these studies as likely substrates for MRP2. Moreover, the active transport of oxaliplatin was shown to be inhibited by myricetin^36^. In the current study, we extended these findings to show lower accumulation of platinum and sensitivity to oxaliplatin-induced growth inhibition in HEK293 cells stably transfected to overexpress MRP2, and in human gastrointestinal tumour lines (HepG2 and PANC-1) with endogenous overexpression of MRP2. Inhibition of MRP2 by myricetin or siRNA knockdown increased the accumulation of platinum and sensitivity to oxaliplatin-induced growth inhibition in these MRP2 overexpressing tumour lines. None of the four colorectal cancer cell lines we studied displayed low oxaliplatin accumulation due to MRP2 overexpression but a hepatocellular (HepG2)^37^ and a pancreatic carcinoma (PANC-1) line did. This created an incomplete match in the data presented here between the clinical-gene expression dataset of colorectal cancer patients (Fig. 1) and our experimental work that mainly focussed on the HepG2^37^ and PANC-1 lines that displayed MRP2-mediated transport-resistance to oxaliplatin *in vitro* (Fig. 3). This incomplete match in the presented data was further compounded by no tumour gene expression clinical datasets of hepatocellular or pancreatic carcinoma patients with annotated tumour response to oxaliplatin being available in Oncomine. However, previous studies have found increased MRP2 expression and activity in oxaliplatin-resistant colorectal cancer lines *in vitro*, and that inhibition of MRP2 expression and activity by genetic knockdown, myricetin or other inhibitors, can reverse MRP2-mediated accumulation deficits and resistance to oxaliplatin in colorectal cancer^24–26^.

This study also provided *in vivo* experimental proof of the therapeutic concept that targeting MRP2 could sensitise MRP2-overexpressing human gastrointestinal tumours to the anti-tumour activity of oxaliplatin without increased toxicity. In mice bearing subcutaneous xenografts of the HepG2 tumour line, which had shown MRP2-mediated deficits in oxaliplatin accumulation *in vitro*, oxaliplatin treatment given alone reduced body weight but had no significant antitumour activity. In contrast, when combined with myricetin, oxaliplatin treatment slowed tumour growth and extended the survival of animals without significant increase in toxicity. However, toxicity was measured only by body weight and the major clinical toxicity of oxaliplatin, neurotoxicity could have been increased by inhibition of MRP2. Further studies will be required to determine if MRP2 inhibition or genetic knockout increases oxaliplatin neurotoxicity, for example, using histomorphometric endpoints (eg. dorsal root ganglion neuron cell body size) of oxaliplatin neurotoxicity^38,39^. While other mechanisms cannot be ruled out^26,40–42^, it is reasonable on the basis of the findings reported here to hypothesise that myricetin sensitises tumours to oxaliplatin antitumour activity by inhibiting oxaliplatin transport mediated by MRP2. In another recent study of an animal model of human gastrointestinal cancer, myricetin enhanced the *in vivo* antitumour activity of 5-fluorouracil^43^, which is often given with oxaliplatin in combination regimens such as FOLFOX. These findings now set the scene for further exploration of the therapeutic concept of targeting MRP2 with myricetin or other inhibitors to enhance the antitumour activity of oxaliplatin-based combination regimens, both experimentally and in the clinic.

In conclusion, MRP2 limits oxaliplatin accumulation and responses in gastrointestinal cancer. As tumour MRP2 expression is both readily measurable and potentially targetable in gastrointestinal cancer patients, screening MRP2 tumour expression to select patients for treatment with oxaliplatin alone or in combination with an MRP2 inhibitor could improve their outcomes.

## Materials and Methods

### Oncomine dataset and analysis

Log2 median-centred intensity values for the tumour expression of eighteen oxaliplatin transporter candidate genes from 83 patients in the Tsuji Colorectal dataset (GDS4393 and GDS4396)^29^ were exported from Oncomine. Patients were stratified according to their response to FOLFOX chemotherapy. Differences between the responder (n = 42) and non-responder (n = 41) groups were analysed by Two-way ANOVA (Two-way ANOVA factors: transporter gene expression and tumour response to FOLFOX) followed by Bonferroni post-tests for each candidate gene. Data were assessed visually in graphs of absolute differences and standard errors of differences in mean log2 median-centred intensity values for each gene between the responder and non-responder groups.

### Drugs and Reagents

Oxaliplatin (Actavis, New Zealand) solution at 5 mg/mL was prepared by dissolving 100 mg powder into 20 mL MilliQ water or 5% glucose. The stock solutions were stored frozen at −20 °C. One month after preparation, the stock solutions were discarded. The cell culture media RPMI 1640, Opti-MEM and stealth RNAi siRNA were from Invitrogen, and Dulbecco’s Modified Eagle Medium (DMEM), and foetal bovine serum (FBS) were from Life Technologies (Auckland, NZ). Myricetin was from Cayman Chem (MI, USA). All other chemicals were purchased from Sigma-Aldrich (St Louis, MO, USA).

### Cell Lines and Tissue Culture

Two isogenic HEK293 (Human Embryonic Kidney-293) sublines, one parental line (HEK-P) and another transfected to stably over-express MRP2 (HEK-MRP2), were kindly provided by Professor Piet Borst (Division of Molecular Biology and Centre for Biomedical Genetics, the Netherlands Cancer Institute, Amsterdam, Netherlands). Apart from the WiDR line that was from Dr Martin Ford (Glaxo-Wellcome, Stevenage, UK), the cell lines were purchased from the American Type Culture Collection (ATCC, Manassas, VA, USA). Cell lines were grown and maintained in Dulbecco’s Modified Essential Medium (DMEM), supplemented with 10% FBS and 100 units of penicillin/streptomycin per ml, at 37 °C under 5% CO2 with 95% humidified air, except for the HepG2 cell line that was grown in Roswell Pack Memorial Institute (RPMI)−1640 or Eagle’s Minimum Essential Medium (MEM) supplemented with 10% (v/v) foetal bovine serum, 2 mmol/L L-glutamine, 100 units/mL penicillin and 100 units/mL streptomycin, in a humidified atmosphere of 5% carbon dioxide at 37 °C. The authenticity of the cell lines was confirmed by short tandem repeat DNA profiling (DNA Diagnostics, Auckland, New Zealand).

### MRP2 Fluorescence Immunocytochemistry

Fluorescent immunocytochemistry was undertaken to detect cellular MRP2 protein expression in the HEK-293 sublines using a primary antibody, anti-MRP2 (1:100; ab3373 from Abcam), and a secondary antibody, Alexa Fluor 594-labeled anti-mouse IgG (Invitrogen, Carlsbad, CA, USA). Cells were cultured in the chamber slides until they reached the confluence of 60–70%. The growth medium was then replaced with pre-warmed PBS which was left for 5 min followed by fixation of cells with 4% paraformaldehyde for 15 min at room temperature. Then, cells were permeabilised with 0.2% Triton X-100 in PBS for 15 min. Blocking of the non-specific protein binding was achieved by incubating cells with blocking solution (PBS containing 0.2% Triton X-100, 3% goat serum, and 2% bovine serum albumin) for 1 h at room temperature. After blocking, cells were incubated with the primary antibody, anti-MRP2 (ab3373 from Abcam) prepared in immunobuffer (PBS containing 0.2% Triton X-100 and 3% goat serum) with the dilution ratio of 1:100 for overnight at 4 °C. After that, the cells were washed for 3–4 times with PBS containing 0.2% Triton X-100 followed by incubation of cells with the secondary antibody (Alexa Fluor 594-labeled anti-mouse IgG, Invitrogen, Carlsbad, CA, USA) diluted in the immunobuffer (dilution ratio was 1:500) for 3 h at 4 °C with the cover to protect from light. Cells were then washed with PBS and cover-slipped with VECTASHIELD Mounting Medium with DAPI (6-diamidino-2-phenylindole) to stain nucleic acid (Vector Laboratories, Burlingame, CA, USA). Images were captured using an Olympus FV1000 confocal laser scanning microscope (Olympus Inc., Tokyo) attached to a Nikon digital camera and then analysed using Nikon Elipse Net (Nikon, Melville, NY, USA) and ImageJ software (National Institutes of Health, Bethesda, MD, USA).

### Platinum accumulation

Inductively coupled plasma mass spectrometry (ICP-MS) was used to measure the cellular content of platinum after exposure to oxaliplatin. For these experiments, cells were seeded at 250,000 cells per well (350,000 cells per well for HepG2 and PANC-1) in collagen-coated six-well plates, and grown in the normal growth medium until around 80% confluent. Cells were then incubated with oxaliplatin at designated concentrations and treatment durations. At the end of the treatment period, cells were washed three times with 1 mL of ice-cold PBS (phosphate buffered saline) then dried in room air for 30 min. Then 330 μL 70% nitric acid was added to each well and the plate gently shaken at room temperature for 2 hours to digest the cells. A 300 μL aliquot of the digest was then added to a 96-well plate to determine protein content using a modified tyrosine nitration assay^44^. After the protein assay, aliquots were transferred to 5 mL screwtop vials for further digestion in 70% nitric acid at room temperature overnight before heating at 95 °C for 2 hours. Digests were diluted in milliQ water containing thallium 50 ppb as an internal standard before ICP-MS analysis using a Varian 820MS ICP-MS (Agilent Technologies Inc., Santa Clara, CA, USA) at LabPLUS (Auckland, New Zealand). Optimisation of signal/noise was carried prior to each run using a tuning solution of platinum and thallium. Platinum isotopes (m/z 194 and 195) and thallium (m/z 205) were monitored during the run. Platinum counts for samples and standards were normalized for thallium internal standard counts. The platinum content of each experimental sample was calculated from the platinum to thallium count ratios and a calibration curve made from standard solutions at desired platinum concentrations made up in the same matrix as the samples. Quality control samples at three different concentrations were included for determing the accuracy, precision and reliability of each run. An ICP-MS analysis run was accepted if the standard curve was linear, and quality control and calibration standards were ±15% of their nominal value and replicate quality control samples had % coefficient of variation of ≤15%. The limit of detection and lower limit of quantification were 0.3 ppb and 1 ppb, respectively.

### Cell growth inhibition

To evaluate the sensitivity of different cell lines to oxaliplatin-induced cell growth inhibition, cells were exposed to oxaliplatin at different concentrations for 2 h followed by measurement of viable cells with MTT assay^45^. Cells were seeded at 5000 cells/well in a collagen-coated 96-well plates then allowed to attach to the well surface over 24 h incubation in normal drug-free growth medium. After attachment, cells were exposed to oxaliplatin at different concentrations for the designated time at 37 °C with 5% CO_2_/95% air. When myricetin was used, cells were exposed to myricetin for 30 min immediately prior to and during the 2 h exposure to oxaliplatin. To terminate drug exposure, the medium containing drug was removed and replaced with the drug-free growth medium. Cells were then allowed to grow for 72 hours under usual incubation conditions before the MTT assay was performed. Percent cell viability at different drug concentrations was calculated from 540 nm absorbance values normalised to the mean absorbance of control untreated cells. IC-50 values for oxaliplatin-induced growth inhibition were determined from the data using nonlinear regression in GraphPad Prism version 6.

### MRP2 function

Cellular 5(6)-carboxy-2,′7′-dichlorofluorescein (CDCF) accumulation in HEK-P, HEK-MRP2 and HepG2 cell lines was measured to evaluate the transporter activity of MRP2^46^. Cells were seeded at the density of 50,000 cells per well (80,000 cells for HepG2) in the collagen coated 24-well plates. Once cells reached 80–90% confluence, they were incubated with 10 μM 5(6)-carboxy-2,′7′-dichlorofluorescein diacetate (CDCFDA) dissolved in 10 mM HEPES-HBSS medium for 90 min at 37 °C. Cells were then washed twice with 10 mM HEPES-HBSS medium, then treated with 0.1% Triton dissolved in 10 mM HEPES-HBSS medium for 10 min at room temperature. Cell lysate samples were then collected in black 96-well plates for the reading of fluorescence intensity using fluorimeter at 485/528 nm with 20 nm wavelength. Protein levels of samples were measured using a modified tyrosine nitration assay^44^ after the cells were digested with 70% nitric acid for 2 hr. The cellular accumulation of CDCF in fluorescence units (FU) per mg of protein was calculated using the fluorescence intensity of lysate measured as arbitrary fluorescence units and the amount of protein of cell lysate measured as mg of protein.

### Real-time Transporter PCR array analysis

Total RNA from cultured cells was extracted using an RNeasy Mini Kit (QIAGEN, Venlo, Netherlands) according to the manufacturer’s instructions. Total RNA content was determined by measuring the absorbance at 260 nm with a NanoDrop ND-1000 spectrophotometer (Thermo Fisher Scientific). cDNA was synthesised from 400 ng RNA using an RT^2^ First Strand Kit (QIAGEN) according to the manufacturer’s instructions, and then mixed with RT^2^ SYBR Green qPCR Mastermix (QIAGEN). The mixtures were loaded into a commercially-available pre-validated RT^2^ Profiler PCR Array 96-well plate containing optimized primer assays for a set of five reference genes (*GUSB, HPRT, HSP90AB1, GAPDH* and *ACTB*) and 86 *ABC* and *SLC* drug transporter genes (QIAGEN). Real-time PCR reactions were performed according to the array manufacturer’s instructions using Applied Biosystems 7900HT Fast Real-Time PCR System and SDS 2.3 software (Life Technologies) repeated three times for each cell line. The temperature profile was as follows: 95 °C for 10 min, and then 40 cycles of 95 °C for 15 s and 60 °C for 1 min. The threshold cycle (Ct) values were obtained for each sample. As previously^47,48^, the expression of the five housekeeping genes varied widely between the different cell lines and were unsuitable for use as controls, and expression level Ct values were normalised by the mean expression of all genes of each cell line and PCR run, then multiplied by −1 so that positive values indicated higher expression. Cell lines were stratified into two groups according to whether they had high or low accumulation of platinum after exposure to oxaliplatin. Differences in transporter gene expression between the groups of cell lines were analysed by Two-way ANOVA (Two-way ANOVA factors: transporter gene expression and platinum accumulation) followed by Bonferroni post-tests for each candidate gene. Data were assessed visually in graphs of absolute differences and standard errors of differences in the expression of each gene between the high and low platinum accumulation groups of cell lines.

### Transfections of siRNA

siRNA targeting the *ABCC2* gene was transfected into HepG2 cells using Invitrogen transfection reagent. A non-targeting negative stealth siRNA (scrambled) was used as a negative control. The cells were seeded at a density of 1.5 × 10^5^ cells per well in 12-well plates. Cells were transfected with different *ABCC2* siRNA subtypes (siRNA-1, siRNA-2 and siRNA-3) at 60 pmol using lipofectamine RNAiMAX (Invitrogen) in Opti-MEM I Reduced Serum Medium (Invitrogen). At 24–96 hr after transfection, experiments were done to assess effects on MRP2 expression and function, platinum accumulation and oxaliplatin-induced growth inhibition. Quantitative real-time PCR was performed to confirm the specific gene silencing by using LightCycler-FastStart DNA Master SYBR Green 1 Master Mix (Roche Applied Science) and gene-specific primers. Total RNA was isolated from cultured cells using RNeasy Mini kit (Qiagen, Valencia, CA) following the manufacturer’s instructions. The quantity of total RNA was measured using Qubit® 2.0 Fluorometer (Invitrogen). Thereafter, cDNA was synthesised from total RNA using Transcriptor First Strand cDNA Synthesis Kit (Roche Applied Science) according to the kit protocol. Primer sequences (MRP2 forward, 5′-AATCAGAGTCAAAGCCAAGATGCC-3′ and reverse, 5′- TAGCTTCAGTAGGAATGATTTCAGGAGCAC-3′; GAPDH forward, 5′- GCACCGTCAAGGCTGAGAAC-3′ and reverse, 5′- GCCTTCTCCATGGTGGTGAA-3′) used to detect the expression of targeted genes were purchased from IDT (Integrated DNA Technology, Singapore). Quantitative real-time PCR was performed with a LightCycler 480 Instrument II (Roche Diagnostics, New Zealand) using LightCycler-FastStart DNA Master SYBR Green 1 Master Mix (Roche Diagnostics, New Zealand) and gene-specific primers at 180 nM. The reaction conditions were as follows: 95 °C for 10 min, followed by 45 cycles at 95 °C for 15 s, at 58 °C for 30 s, and 72 °C for 30 s. The results were analysed using comparative threshold cycle method. Accumulation of an MRP2 specific substrate, 5(6)-carboxy-2′,7′-dichlorofluorescein (CDCF) by transfected cells were performed by incubating cells of density 800,000 cells/mL with 5 µM of CDCFDA for 20 min at 37 °C. After the incubation, cells were washed twice, re-suspended in ice-cold PBS and kept at 4 °C until analysis by flow cytometry (Beckman Coulter MoFlow XDP) with standard laser for excitation at 488 nm and band pass filter at 525 nm. Cells were gated to exclude dead cells, cellular debris and cell doublets. The mean fluorescence intensity was measured using Kaluza software (Beckman Coulter). Effects of siRNA transfection on cellular accumulation of platinum and oxaliplatin-induced growth inhibition was determined as described above.

### Animal experiments

Animals were housed in a temperature-controlled environment with access to food and water *ad libitum*, and were acclimatized to handling prior to the experiments. Pilot studies were undertaken to establish the treatment protocol prior to the definitive experiment. The pilot studies showed similar trends as shown in the final experiment. For the definitive experiment, 32 NIH-III nude mice weighing 18 to 21 g were injected subcutaneously into the right flank with 10^7^ of HepG2 cells in three batches of 10 or 11 animals each. When tumours measured approximately 200 mm^3^ (range, 161 to 237 mm^3^), animals were randomly allocated to one of four treatment groups (control, oxaliplatin alone, myricetin alone or oxaliplatin plus myricetin). Accordingly, animals were treated with oxaliplatin (3 mg/kg ip), myricetin (25 mg/kg iv) or matching drug vehicle, alone or in combination once weekly for up to five weeks. Oxaliplatin was given 30 mins after myricetin or its vehicle. Animals were monitored during the treatment period twice each week by general inspection, and measurement of body weight and perpendicular tumour dimensions using calipers. Animals were euthanased if they had poor general condition, greater than 15% body weight reduction or had tumours greater than 20 mm in average diameter or 10% of body weight, with ulceration or restricting movement. Body weight was expressed as percentage of baseline. Tumour volumes were calculated using the formula π/6*w*l^2^, where w = the longest diameter and l = the shortest diameter. Change in body weight and tumour volume from baseline was assessed visually by plotting mean and standard error of the mean versus time, and analysed by Two-way Repeated Measures ANOVA with Bonferroni post-tests. Time to event endpoints (quadrupling of tumour volume and euthanasia) were assessed by Kaplan-Meier plots and analysis by Gehan-Breslow-Wilcoxon test without adjustment for multiple testing. All animal procedures were approved by and performed in compliance with ethical guidelines of the Animal Ethics Committee of the University of Auckland.

### Data analysis

Data were analysed using GraphPad Prism 6.2 software (San Diego, CA, USA). *In vitro* experiments were replicated at least three times. The significance of differences between means was assessed by ANOVA and post-tests with correction for repeated testing, as appropriate for the data. For repeated measures ANOVA, missing values (3 out of a total of 64 values (<5%)) were replaced by imputation. Differences in time to event (survival) curves were analysed using the Gehan-Breslow-Wilcoxon test without adjustment for multiple testing. A *P* < 0.05 was regarded as statistically significant for all statistical tests.

## Supplementary information

Supplementary tables

## Data Availability

The datasets generated during and analysed during the current study are available from the corresponding author on reasonable request.
