# Supplementary material for: Identification of MRP2 as a targetable factor limiting oxaliplatin accumulation and response in gastrointestinal cancer
Source: Sci Rep. 2019 Feb 19;9:2245. doi: 10.1038/s41598-019-38667-8 (PMC6381153; doi:10.1038/s41598-019-38667-8)
Supplement: Supplementary file 1 — Supplementary tables [file 41598_2019_38667_MOESM1_ESM.pdf]

Identification of MRP2 as a targetable factor limiting oxaliplatin accumulation and response in gastrointestinal cancer

Khine Myint[1], Riya Biswas[2], Yan Li[2,3], Nancy Jong[1], Stephen Jamieson[1,4], Johnson Liu[5], Catherine Han[1,4], Christopher Squire[6], Fabrice Merien[2], Jun Lu[2,3], Takeo Nakanishi[7], Ikumi Tamai[7], Mark McKeage[1,4]

Affiliations:

1 Department of Pharmacology and Clinical Pharmacology, University of Auckland, Auckland, New Zealand;

2 AUT-Roche Diagnostics Laboratory, School of Science, Auckland University of Technology, Auckland, New Zealand;

3 School of Interprofessional Health Studies, Auckland University of Technology, Auckland, New Zealand;

4 Auckland Cancer Society Research Centre, University of Auckland, Auckland, New Zealand;

5 Department of Pharmacology, School of Medical Sciences, University of New South Wales, Sydney, NSW 2052, Australia

6 School of Biological Sciences, University of Auckland, Auckland, New Zealand;

7 Department of Membrane Transport and Biopharmaceutics, Faculty of Pharmaceutical Sciences, Institute of Medical, Pharmaceutical and Health Sciences, Kanazawa University, Kakuma-machi, Kanazawa 920-1192, Japan

Correspondence: Professor Mark McKeage, Department of Pharmacology and Clinical Pharmacology and Auckland Cancer Society Research Centre, School of Medical Sciences, Faculty of Medical and Health Sciences, University of Auckland, Private Bag 92129 Auckland, New Zealand; email m.mckeage@auckland.ac.nz; tel +6493737599 x87322; Fax +6499347584

Supplementary table 1 Two-way repeated measures ANOVA multiple comparisons for body weight

Day	Bonferroni's multiple comparisons test	Mean Diff.	95.00% CI of diff.	Significant?	Summary	Adjusted P Value
0	Myricetin alone vs. Control	0	-3.803 to 3.803	No	ns	>0.9999
	Oxaliplatin alone vs. Control	0	-3.803 to 3.803	No	ns	>0.9999
	Combination vs. Control	0	-3.803 to 3.803	No	ns	>0.9999
	Oxaliplatin alone vs. Myricetin alone	0	-3.803 to 3.803	No	ns	>0.9999
	Combination vs. Myricetin alone	0	-3.803 to 3.803	No	ns	>0.9999
	Combination vs. Oxaliplatin alone	0	-3.803 to 3.803	No	ns	>0.9999
3	Myricetin alone vs. Control	1.483	-2.32 to 5.285	No	ns	>0.9999
	Oxaliplatin alone vs. Control	-1.05	-4.853 to 2.753	No	ns	>0.9999
	Combination vs. Control	-2.496	-6.299 to 1.306	No	ns	0.4916
	Oxaliplatin alone vs. Myricetin alone	-2.533	-6.335 to 1.27	No	ns	0.4657
	Combination vs. Myricetin alone	-3.979	-7.781 to -0.1761	Yes	*	0.0348
	Combination vs. Oxaliplatin alone	-1.446	-5.249 to 2.356	No	ns	>0.9999
7	Myricetin alone vs. Control	0.4488	-3.354 to 4.251	No	ns	>0.9999
	Oxaliplatin alone vs. Control	-1.694	-5.496 to 2.109	No	ns	>0.9999
	Combination vs. Control	-4.064	-7.866 to -0.2611	Yes	*	0.0291
	Oxaliplatin alone vs. Myricetin alone	-2.143	-5.945 to 1.66	No	ns	0.8105
	Combination vs. Myricetin alone	-4.513	-8.315 to -0.7099	Yes	*	0.0108
	Combination vs. Oxaliplatin alone	-2.37	-6.173 to 1.433	No	ns	0.591
10	Myricetin alone vs. Control	-0.585	-4.388 to 3.218	No	ns	>0.9999
	Oxaliplatin alone vs. Control	-3.231	-7.034 to 0.5714	No	ns	0.148
	Combination vs. Control	-4.484	-8.286 to -0.6811	Yes	*	0.0116
	Oxaliplatin alone vs. Myricetin alone	-2.646	-6.449 to 1.156	No	ns	0.3917
	Combination vs. Myricetin alone	-3.899	-7.701 to -0.09613	Yes	*	0.0411
	Combination vs. Oxaliplatin alone	-1.253	-5.055 to 2.55	No	ns	>0.9999
14	Myricetin alone vs. Control	0.9063	-2.896 to 4.709	No	ns	>0.9999
	Oxaliplatin alone vs. Control	-1.299	-5.101 to 2.504	No	ns	>0.9999
	Combination vs. Control	-2.273	-6.075 to 1.53	No	ns	0.6784
	Oxaliplatin alone vs. Myricetin alone	-2.205	-6.008 to 1.598	No	ns	0.7447
	Combination vs. Myricetin alone	-3.179	-6.981 to 0.6239	No	ns	0.1624
	Combination vs. Oxaliplatin alone	-0.9738	-4.776 to 2.829	No	ns	>0.9999
17	Myricetin alone vs. Control	-0.00625	-3.809 to 3.796	No	ns	>0.9999
	Oxaliplatin alone vs. Control	-3.216	-7.019 to 0.5864	No	ns	0.152
	Combination vs. Control	-3.91	-7.713 to -0.1074	Yes	*	0.0402
	Oxaliplatin alone vs. Myricetin alone	-3.21	-7.013 to 0.5926	No	ns	0.1537
	Combination vs. Myricetin alone	-3.904	-7.706 to -0.1011	Yes	*	0.0407
	Combination vs. Oxaliplatin alone	-0.6938	-4.496 to 3.109	No	ns	>0.9999
21	Myricetin alone vs. Control	-1.593	-5.395 to 2.21	No	ns	>0.9999
	Oxaliplatin alone vs. Control	-2.578	-6.38 to 1.225	No	ns	0.4352
	Combination vs. Control	-4	-7.803 to -0.1974	Yes	*	0.0333
	Oxaliplatin alone vs. Myricetin alone	-0.985	-4.788 to 2.818	No	ns	>0.9999
	Combination vs. Myricetin alone	-2.408	-6.21 to 1.395	No	ns	0.56
	Combination vs. Oxaliplatin alone	-1.423	-5.225 to 2.38	No	ns	>0.9999
24	Myricetin alone vs. Control	-1.814	-5.616 to 1.989	No	ns	>0.9999
	Oxaliplatin alone vs. Control	-3.42	-7.223 to 0.3826	No	ns	0.1049
	Combination vs. Control	-5.454	-9.256 to -1.651	Yes	**	0.001
	Oxaliplatin alone vs. Myricetin alone	-1.606	-5.409 to 2.196	No	ns	>0.9999
	Combination vs. Myricetin alone	-3.64	-7.443 to 0.1626	No	ns	0.069
	Combination vs. Oxaliplatin alone	-2.034	-5.836 to 1.769	No	ns	0.9356

Supplementary table 2 Two-way repeated measures ANOVA multiple comparisons for tumour volume

Day	Bonferroni's multiple comparisons test	Mean Diff.	95.00% CI of diff.	Significant?	Summary	Adjusted P Value
0	Control vs. Myricetin alone	0	-167.8 to 167.8	No	ns	>0.9999
	Control vs. ox alone	0	-167.8 to 167.8	No	ns	>0.9999
	Control vs. combination	0	-167.8 to 167.8	No	ns	>0.9999
	Myricetin alone vs. ox alone	0	-167.8 to 167.8	No	ns	>0.9999
	Myricetin alone vs. combination	0	-167.8 to 167.8	No	ns	>0.9999
	ox alone vs. combination	0	-167.8 to 167.8	No	ns	>0.9999
3	Control vs. Myricetin alone	-13.83	-181.6 to 154	No	ns	>0.9999
	Control vs. ox alone	13.08	-154.7 to 180.9	No	ns	>0.9999
	Control vs. combination	33.59	-134.2 to 201.4	No	ns	>0.9999
	Myricetin alone vs. ox alone	26.91	-140.9 to 194.7	No	ns	>0.9999
	Myricetin alone vs. combination	47.42	-120.4 to 215.2	No	ns	>0.9999
	ox alone vs. combination	20.51	-147.3 to 188.3	No	ns	>0.9999
7	Control vs. Myricetin alone	-72.29	-240.1 to 95.5	No	ns	>0.9999
	Control vs. ox alone	18.91	-148.9 to 186.7	No	ns	>0.9999
	Control vs. combination	59.02	-108.8 to 226.8	No	ns	>0.9999
	Myricetin alone vs. ox alone	91.21	-76.59 to 259	No	ns	0.8959
	Myricetin alone vs. combination	131.3	-36.49 to 299.1	No	ns	0.2303
	ox alone vs. combination	40.1	-127.7 to 207.9	No	ns	>0.9999
10	Control vs. Myricetin alone	-119.7	-287.4 to 48.14	No	ns	0.3538
	Control vs. ox alone	16.53	-151.3 to 184.3	No	ns	>0.9999
	Control vs. combination	78.77	-89.03 to 246.6	No	ns	>0.9999
	Myricetin alone vs. ox alone	136.2	-31.61 to 304	No	ns	0.1908
	Myricetin alone vs. combination	198.4	30.62 to 366.2	Yes	*	0.0112
	ox alone vs. combination	62.24	-105.6 to 230	No	ns	>0.9999
14	Control vs. Myricetin alone	-117.1	-284.9 to 50.69	No	ns	0.3871
	Control vs. ox alone	-24.7	-192.5 to 143.1	No	ns	>0.9999
	Control vs. combination	109.6	-58.22 to 277.4	No	ns	0.5012
	Myricetin alone vs. ox alone	92.41	-75.39 to 260.2	No	ns	0.8644
	Myricetin alone vs. combination	226.7	58.88 to 394.5	Yes	**	0.0024
	ox alone vs. combination	134.3	-33.52 to 302.1	No	ns	0.2055
17	Control vs. Myricetin alone	-192.7	-360.5 to -24.95	Yes	*	0.015
	Control vs. ox alone	-92.33	-260.1 to 75.46	No	ns	0.8663
	Control vs. combination	127.2	-40.57 to 295	No	ns	0.2685
	Myricetin alone vs. ox alone	100.4	-67.38 to 268.2	No	ns	0.6755
	Myricetin alone vs. combination	320	152.2 to 487.8	Yes	****	<0.0001
	ox alone vs. combination	219.6	51.76 to 387.4	Yes	**	0.0036
21	Control vs. Myricetin alone	-207.3	-375.1 to -39.55	Yes	**	0.007
	Control vs. ox alone	-97.44	-265.2 to 70.35	No	ns	0.7414
	Control vs. combination	126	-41.83 to 293.8	No	ns	0.2814
	Myricetin alone vs. ox alone	109.9	-57.89 to 277.7	No	ns	0.4957
	Myricetin alone vs. combination	333.3	165.5 to 501.1	Yes	****	<0.0001
	ox alone vs. combination	223.4	55.61 to 391.2	Yes	**	0.0029
24	Control vs. Myricetin alone	-105.3	-273.1 to 62.46	No	ns	0.5766
	Control vs. ox alone	-165.5	-333.3 to 2.336	No	ns	0.0556
	Control vs. combination	188.7	20.86 to 356.5	Yes	*	0.0184
	Myricetin alone vs. ox alone	-60.12	-227.9 to 107.7	No	ns	>0.9999
	Myricetin alone vs. combination	294	126.2 to 461.8	Yes	****	<0.0001
	ox alone vs. combination	354.1	186.3 to 521.9	Yes	****	<0.0001

Supplementary table 3 Results of multiple comparisons for time to event endpoints .

Comparison	P values (unadjusted)	
	Time to euthanasia	Time for tumours to quadruple in volume
All groups	**0.0032**	**0.0112**
Control versus myricetin alone	0.0989	0.1776
Control versus oxaliplatin alone	0.0848	0.1278
Oxaliplatin alone versus myricetin alone	0.1217	0.6605
Combination versus control	0.0968	**0.0454**
Combination versus myricetin alone	**0.0181**	**0.0150**
Combination versus oxaliplatin alone	**0.0003**	**0.0016**
